# Induction of a robust immunity response against novel duck reovirus in ducklings using a subunit vaccine of sigma C protein

**DOI:** 10.1038/srep39092

**Published:** 2016-12-15

**Authors:** Zhuangli Bi, Yingqi Zhu, Zongyan Chen, Chuanfeng Li, Yong Wang, Guijun Wang, Guangqing Liu

**Affiliations:** 1 Shanghai Veterinary Research Institute, Chinese Academy of Agricultural Sciences, Shanghai 200241, China; 2 College of Animal Science and Technology, Anhui Agricultural University, Hefei 230036, China

## Abstract

Novel duck reovirus (NDRV) disease emerged in China in 2011 and continues to cause high morbidity and about 5.0 to 50% mortality in ducklings. Currently there are no approved vaccines for the virus. This study aimed to assess the efficacy of a new vaccine created from the baculovirus and sigma C gene against NDRV. In this study, a recombinant baculovirus containing the sigma C gene was constructed, and the purified protein was used as a vaccine candidate in ducklings. The efficacy of sigma C vaccine was estimated according to humoral immune responses, cellular immune response and protection against NDRV challenge. The results showed that sigma C was highly expressed in Sf9 cells. Robust humoral and cellular immune responses were induced in all ducklings immunized with the recombinant sigma C protein. Moreover, 100% protection against lethal challenge with NDRV TH11 strain was observed. Summary, the recombinant sigma C protein could be utilized as a good candidate against NDRV infection.

Avian orthoreovirus (ARV) is an important etiological agent that can cause several economically devastating diseases in a variety of domestic and wild birds, including chickens^1^, turkeys^2^, Muscovy ducks^3^, Pekin ducks^4^, geese^5^,^6^, wild mallard ducks^7^, pigeons^8^, psittacine birds^9^ and other wild birds. In waterfowl species, causative ARVs were first isolated in France in 1972 from Muscovy ducks, historically termed Muscovy duck reovirus (MDRV)^3^ and emerged in China in 1997^10^. MDRV infects mainly Muscovy ducklings at 10 d old and can persist in an infected flock until the ducklings are 6.0 weeks old, with a mortality rate of from 10 to 50%^3^,^11^,^12^. The clinical signs are diarrhea, enteritis, weight loss, serofibrinous pericarditis, gouty kidneys, and the inability to walk and post-mortem examinations show a swollen and hemorrhagic liver and spleen covered with small white necrotic foci^3^,^10^,^12^. However, it has been reported that MDRV is non-pathogenic for shelduck ducklings, Pekin ducklings, or other varieties of ducklings^10^,^13^.

In recent years, a novel duck reovirus (NDRV) disease, called “spleen necrosis disease”, “new liver disease in Muscovy ducks” or “duck hemorrhagic-necrotic hepatitis”, was commonly found among the young of different duck species, such as shelducks, Pekins, wild mallards, and Muscovy, and also in goslings in some parts of China^7^,^14^,^15^,^16^. The disease is characterized mainly by severe hemorrhagic-necrotic lesions in the liver and spleen, which distinguishes it from an MDRV infection, and the mortality rate is ~5.0 to 50%. A similar ARV infection was reported in southwest Poland in 2012^17^. Of greater concern is that the current commercial vaccines against ARVs or MDRVs do not completely prevent NDRV infection and transmission. Therefore, it is crucial that a safe and efficient vaccine against NDRV infection be developed as soon as possible.

The ARV genome consists of 10 double-stranded (ds) RNA segments that are divided into three size classes, large (L), medium (M) and small (S) according to their electrophoretic mobility^18^. Sigma C protein, which is encoded by the S1 segment, is a compartment of the outer capsid of the virion and is the main cell attachment protein of ARV^19^. Some studies have suggested that sigma C is the most variable of all ARV proteins^20^ and is the only target for type-specific neutralizing antibodies^21^,^22^. Therefore, sigma C is the leading target in the development of a subunit vaccine against ARV infection, although the immunogenicity of NDRV sigma C has not yet been reported. The sigma C protein of NDRV is structurally similar to the sigma C protein of the known ARV^23^.Therefore, we presumed that NDRV sigma C might be functionally related to ARV sigma C and plays an important role in the immune response against NDRV infection.

The baculovirus expression system has been used widely as an excellent tool by which to express recombinant proteins as subunit vaccines. The system has two distinct advantages over other expression systems as follows: the recombinant proteins can be expressed at high levels and the proteins can be maintained in both their natural antigenicity and immunogenicity^24^,^25^,^26^. In the present study, a recombinant NDRV sigma C protein fused with a 6 X His tag was expressed in the baculovirus expression system. The vaccine potential of the purified recombinant sigma C protein was further evaluated its in terms of the humoral and cell-mediated immune responses as well as protection against the NDRV challenge in ducklings.

## Materials and Methods

### Ethics Statement

We confirmed that all animal experiments were carried out in accordance with the guidelines of the Guide for the Care and Use of Laboratory Animals of the Institutional Animal Care and Use Committee (IACUC) set by Shanghai Veterinary Research Institute, the Chinese Academy of Agricultural Sciences. The protocols were approved by the Animal Care and Use Committee of Shanghai Veterinary Research Institute (Shanghai, China). The birds were treated humanely, and all experiments were taken under minimize suffering conditions. Ducklings were monitored every 12 hours over a period of 6 weeks for health and signs of disease. We used aspirin to minimize animal suffering and distress after virus challenge. A humane endpoint was used in our experiments. The specific signs used to determine the endpoint include: a). Loss of >20% body weight compared with the starting weight; b). Decreased food or water intake; c). Showed clinical signs of diarrhea, depression, lameness and paralysis. To ameliorate suffering, sodium pentobarbital were used for euthanasia in this study.

### Cells and Virus

BHK-21 cells were purchased from Cell Bank of Chinese Academy of Sciences (Shanghai, China), and cultured in Dulbecco’s modified Eagle medium (DMEM; Gibco) supplemented with 10% fetal bovine serum (FBS; Gibco), 100U/ml penicillin and 100 μg/ml streptomycin, and incubated at 37 °C under 5% CO_2_. *Spodoptera frugiperda* 9 (Sf9) cells were grown and maintained in monolayer cultures at 28 °C using serum-free Sf-900^TM^ II SFM (1×) (Gibco, Grand Island, NY). The novel duck reovirus strain TH11 (NDRV-TH11) was recently isolated from Pekin ducklings (*Anas platyrhynchos*) in the southeastern China^16^ and were propagated in SPF duck embryos. The virus was cloned and titrated on fertile duck eggs. Virus titers were expressed as log_10_ 50% embryo lethal dose (ELD_50_).

### Construction of recombinant baculovirus

**Figure 1:**
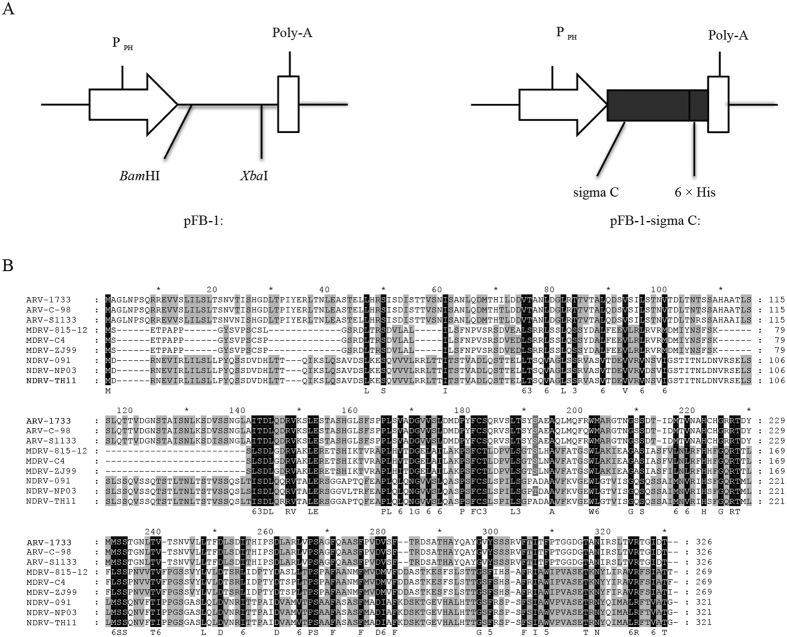
Schematic representation of the structure of pFB-1-sigma C (**A**) and amino acid sequence alignment of the sigma C protein of NDRVs compared with those of ARVs and MDRVs. (**B**). Notes: pFB-1, pFastBac1 vector; P_PH_, the polyhedron promoter of the baculovirus; Poly-A, polyadenylation signal.

The coding region of NDRV sigma C was amplified from NDRV strain TH11 (GenBank: JX826587.1) and inserted under the control of the polyhedrin promoter in the pFastBac1 vector (Invitrogen, USA) (Fig. 1A). Primers used for the construction of the fusion of NARV sigma C protein and 6 X His tag are summarized in Table 1. The PCR amplification was carried out in a 50 μl reaction mixture containing 0.2 μM dNTP, 0.5 mM MgCl_2_, 0.2 μM each primers, 1 × PCR buffer and 1 U of Platinum Taq polymerase (Invitrogen). PCR thermal profile was 94 °C for 3 min followed by 30 cycles at 94 °C for 30 s, 55 °C for 30 s, 72 °C for 100 s, then a final extension step at 72 °C for 5 min.

The amplified fragments were digested in reactions at 37 °C for 3 h with restriction enzymes *Bam*HI and *Xba*I (Takara, China) and purified using the TakaRa PCR Cleaning Kit (Takara, China) then inserted into corresponding regions of pFastBac1 (pFB-1) (Fig. 1A). Identity of the resulting construct, pFB-1-sigma C, was verified by DNA sequencing. The recombinant plasmid was sequenced in both directions using universal pFastBac-C F and pFastBac-C R primers. Sequencing was conducted by Sanggong Biotechnology (Sanggong, China).

The recombinant baculovirus rBv-sigma C was generated using a Bac-to-Bac baculovirus expression system (Invitrogen, USA) according to the manufacturer’s protocol. Briefly, the construct pFB-1-sigma C was integrated into the baculovirus genome within DH10 Bac (Invitrogen, USA) by site-specific transposition. Then, the recombinant bacmid was transfected into Sf9 cells to produce recombinant baculovirus. Finally, the virus was purified by two rounds of plaque assays to obtain higher tiers of viral stocks. The virus titer calculated by plaque assay was 8.5 × 10^8^ PFU.

### Immunofluorescence assays (IFA)

An indirect IFA was used to detect sigma C protein expression in Sf9 cells. Briefly, the cells were fixed in cold methanol: acetone (1: 1, v: v) at −20 °C for 30 min. After three times washed with PBS, cells were incubated at 37 °C for 1 h with mouse antiserum specific for sigma C protein (prepared from Balb/c mouse vaccinated three times with the recombinant NDRV sigma C) diluted 1:500 in PBS. After washing with PBS three times, cells were incubated at 37 °C for 1 h with FITC-conjugated goat anti-mouse IgG antibody (1: 100 dilution with 0.1% Evans blue solution; ZSbio, China). Finaly, the stained cells washed three times with PBS were observed under a fluorescence microscope equipped with a video documentation system (Nikon, Japan).

### SDS–PAGE and Western blot analysis

The recombinant baculovirus infected sf9 cells were collected and lysed in RIPA lysis buffer (Beyotime Biotechnology, China) for 30 min at 4 °C. The lysates were centrifuged for 5 min at 12,000 g at 4 °C. The lysates were further denatured by incubation for 5 min at 95 °C in sample buffer(2% SDS, 10% glycerol, 60 mM Tris (pH 6.8), 5% β-mercaptoethanol, 0.01% bromophenol blue. The samples were then subjected to 12% SDS–PAGE and stained with coomassie blue or were transferred to nitrocellulose (NC) membranes (Amersham, UK) for western blot analysis. To eliminate possible non-specific binding, the membranes were blocked overnight with 10% skim milk (Sigma-Aldrich, USA) in 0.5% Tween 20 in PBS (PBST) for 1 h at room temperature and then incubated with mouse antiserum specific for sigma C protein (diluted 1:500) or mouse anti-6 × His monoclonal antibody (1:1000, Beyotime Biotechnology, China). Horseradish peroxidase (HRP)-conjugated rabbit anti-mouse IgG (ZSbio, China) diluted 1:5000 in PBS was used as the secondary antibody reacted for 1 h at room temperature. Antibody binding was visualised by 3,3′-diaminobenzidine tetrahydrochloride (DAB; Sigma–Aldrich) staining.

### Extensive sigma C protein expression

For expression of sigma C protein, Sf9 cells were infected with the recombinant baculovirus rBv-sigma C at a MOI of 1 and harvested at 72 h post-infection. The expressed fusion protein was purified using Ni-NTA Purification System (Qiagen, Valencia, CA) according to the manufacturer’s protocol. Protein concentration was quantified using an enhanced BCA protein assay kit (Beyotime Biotechnology, China). Finally, purified sigma C protein was filter-sterilized using a 0.2 μm filter and stored at −80 °C.

### Immunization experiments and viral challenge

Forty 1-week-old SPF sheldrake ducklings hatched from SPF sheldrake eggs (Harbin Veterinary Research Institute, China) were divided into four groups (named Bac-sigma C, Bac-wt, NDRV-TH11 strain and PBS) randomly. They were maintained in four separated rooms. Ducklings in Bac-sigma C group were immunized two times subcutaneously at 2-week intervals with purified sigma C protein, in the presence of complete Freund’s adjuvant (Sigma, Louis, MO, USA) for the first inoculation, and incomplete adjuvant for the second immunization. In Bac-sigma C group, ducklings were immunized subcutaneously in the side of the neck with 200 μl Freund’s adjuvant emulsion containing 5 μg of sigma C protein. Similarly, ducklings in group Bac-wt were immunized with 200 μl Freund’s adjuvant emulsion containing 5 μg of lysate of Sf9 cells infected with wild type baculovirus. The lysate were gone through the same purification procedure as the lysate of Sf9 cells infected with the recombinant baculovirus rBv-sigma C. Group ‘NDRV-TH11 strain’ was subcutaneously inoculated with 200 μl of inactivated NDRV vaccine (prepared by our laboratory) and used as positive control. Ducklings in group PBS were received a subcutaneous injection with 200 μl of PBS and served as the mock group. Ducklings of all the vaccinated groups were received an identical booster immunization at 2 weeks post primary immunization. At 0, 7, 14, 21 and 28 days post primary immunization, serum samples were collected to detect the sigma C protein specific antibodies, neutralizing antibodies against NDRV and cell-mediated immune assay. At 14 and 28 days post primary immunization, blood was collected for lymphocyte proliferation assay. Then, ducklings of all groups were challenged intramuscularly with 0.5 ml NDRV-TH11 strain containing 10^6.7^ ELD_50_/ml, 2 weeks after the second immunization. All ducklings were housed in an isolation facility and monitored daily and weighed day about after challenge. All dead ducklings were examined for macroscopic lesions. Two weeks after challenge, the remaining ducklings were humanely euthanized and their blood, liver and spleen samples were collected for detection of NDRV TH11 viral load. The animal experiments were replicated three times and the representative data were shown in this article while the data of the replicate experiments were shown in supplement files.

### Detection of anti-sigma C antibodies

Serum samples from ducklings were determined by an indirect ELISA test using the recombinant sigma C protein of NDRV, produced in E. coli BL21 (DE3), as antigen. The sigma C protein was expressed in E. coli BL21 (DE3) using the PET 32a expression system (Novagen, USA) and the recombinant product was purified by dialysis method. Ninety-six wells flat-bottomed plates (Corning Costar, USA) were coated with recombinant sigma C protein in 0.1 M carbonate/bicarbonate buffer (pH9.6) and incubated overnight at 4 °C. After blocking with 5% BSA in PBS, plates were incubated with duplicate twofold serial dilutions of test sera for 1 h at 37 °C. Rabbit anti-duck IgG HRP (KPL, USA) at a 1:2000 dilution was then added for 1 h at 37 °C, followed by the addition of the substrate 2 mM Sulfuric acid. Absorbance was determined at 450 nm using a Bio-Rad microtitre plate reader.

### Serum neutralization assays

Prior to testing, sera were incubated for 30 min at 56 °C to inactivate complement. Sera of three groups were diluted two-fold serially, mixed with 200 TCID_50_ of NDRV strain TH11 in a 100 μL volume and incubated for 1 h at 37 °C. After incubation, the virus-serum mixtures were injected into the allantoic cavity of 9-day-old SPF duck embryonated eggs. PBS and negative serum were used as negative controls. After 5–7 days of incubation at 37 °C, serum neutralization titers were calculated. Neutralization titers were calculated as the reciprocal of the highest serum dilution that inhibited egg embryo death 7 days post-inoculation.

### Lymphocyte proliferation assay

At 14 and 28 days post immunization, peripheral blood mononuclear cells (PBMCs) were separated from the jugular vein blood of each ducklings using lymphocyte separation medium (Dakewe, Beijing, China). PBMCs resuspended at 1 × 10^6^ cells/ml with complete medium of DMEM containing 10% FBS, were seeded into 96-well plates with 100 μl per well. Subsequently, each sample was stimulated with 100 μl complete medium containing purified sigma C antigen (20 μg/ml) or 100 μl complete medium alone in triplicate. The proliferative activity was measured by a MTT method according to one of our previously study^27^.

### IFN-γ and IL-4 release assays

IFN-γ and IL-4 levels were measured by commercially available duck IFN-γ or IL-4 ELISA kit according to the manufacturer protocols (Shanghai Lengton Bioscience Co.,LTD, Shanghai, China). The concentrations of duck IFN-γ and IL-4 in the serum samples were calculated from a standard curve.

Quantitative real-time RT-PCR (qRT-PCR) for the detection of viral RNA.

Detection of viral load in the tissues of ducklings was performed by qRT-PCR assay referred to our previous study^28^. Total RNA was extracted from the samples (blood, spleen and liver) of ducklings using Trizol reagent (Invitrogen, Carlsbad, CA, USA), and used immediately for qRT-PCR assay using SuperScript III Platinum One-Step qRT-PCR Kit (Invitrogen, Carlsbad, CA, USA). The primers were designed referring to the sequences of sigma C gene of NDRV strain TH11 (Table 1). PCR conditions and cycling parameters are as follows: one cycle at 50 °C for 30 min for the synthesis of cDNA; one cycle at 95 °C for 15 min; 40 cycles at 95 °C for 15 s, 60 °C for 30 s.

### Statistical analysis

The statistical analysis was conducted using Prism version 5.00 software (Graphpad software, California, USA). All data were recorded as means ± SD. The differences of sigma C-specific antibodies, neutralization antibodies, stimulation index reflecting lymphocyte proliferation, cytokine levels, percentages reflecting body weight loss and viral load were evaluated with One-Way ANOVA and Tukey’s multiple comparison tests between groups. A p-value < 0.05 was considered significant. Survival curves were made using the Kaplan-Meier method, and analyzed with a Log-rank (Mantel-Cox) test.

## Results

### Expression and purification of recombinant NDRV sigma C protein

**Figure 2:**
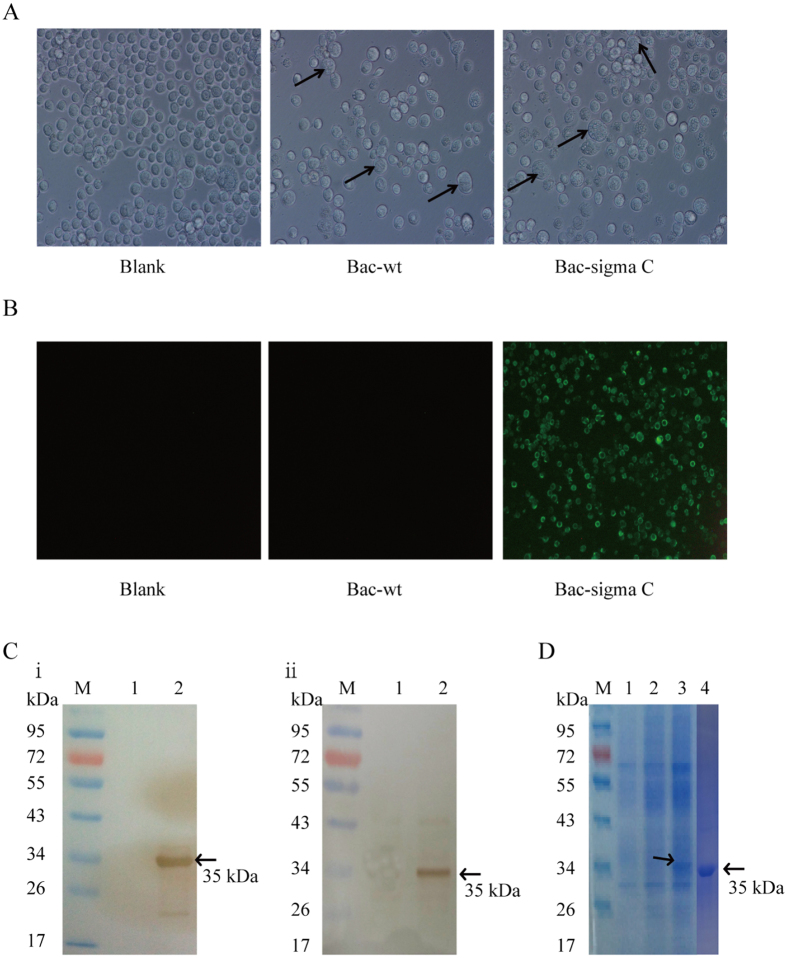
Identification and purification of recombinant sigma C protein. (**A**) Characterization of baculovirus infected cells. Sf9 cells were infected with the recombinant sigma C baculovirus (Bac-sigma C), wild-type baculovirus (Bac-wt), and blank control (Blank). The arrows indicate larger cell size formation in Sf9 cells infected with Bac-wt and Bac-sigma C. (**B**) Immunofluorescence assay (IFA) of Bac-sigma C infected cells. After 48 h post-infection, cells were analyzed by IFA using the mouse antiserum of sigma C as primary antibody and FITC-conjugated goat anti-mouse IgG antibody as secondary antibody (original magnification, 100x). (**C**) Western blot analysis of recombinant sigma C protein in Bac-sigma C infected cells using anti-sigma C serum (i) and anti-His monoclonal antibody (ii). Lane M, molecular weight markers; Lane 1: Sf9 cells infected with Bac-wt; Lane 2: Sf9 cells infected with Bac-sigma C. (**D**) Sodium dodecyl sulfate polyacrylamide gel electrophoresis (SDS-PAGE) analysis of the purified sigma C fusion protein. Lane M, molecular weight markers; Lane 1: non-treated Sf9 cells; Lane 2: Sf9 cells infected with Bac-wt; Lane 3: Sf9 cells infected with Bac-sigma C; Lane 4: 2 μg purified sigma C protein.

NDRV sigma C gene derived from the TH11 strain was expressed in Sf9 cells under the control of the polyhedron promoter using a recombinant baculovirus expression system (Fig. 1A). The Sf9 cells were infected with recombinant baculovirus rBv-sigma C at a multiplicity of infection (MOI) of 1 for the expression of sigma C and infected with wild-type baculovirus as a control. As shown in Fig. 2A, Sf9 cells infected with baculovirus show a typical cytopathic effect (CPE), such as larger size, decreased density, and loose attachment to the culture plate compared to the uninfected control Sf9 cells^29^. The expression of recombinant sigma C was monitored by immunofluorescent assay (IFA) and Western blot analysis. As expected, distinct fluorescent signals were detected in the cells infected with rBV-sigma C but not in either the wild-type baculovirus infected cells or the normal cells (Fig. 2B). In addition, the cells infected with rBV-sigma C expressed proteins with a molecular weight of 35 KD, as identified by both mouse antiserum specific for sigma C protein (Fig. 2Ci) and mouse anti-6 × His monoclonal antibody (Fig. 2Cii). By contrast, no specific protein was detected in wild-type baculovirus infected cells. Next, the recombinant sigma C protein was purified using the Ni-NTA Purification System (Qiagen, Valencia, CA, USA), and analyzed using the Coomassie blue-stained sodium dodecyl sulfate polyacrylamide gel electrophoresis (SDS-PAGE) gels (Fig. 2D). The purified recombinant sigma C protein was also confirmed by Western blot analysis as having high immunoreactivity of the anti-sigma C antibody (data not shown).

### Humoral immune responses in ducklings immunized with recombinant sigma C

**Figure 3:**
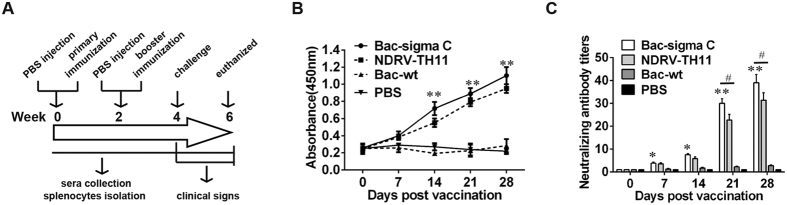
Antibody response in ducklings immunized with recombinant sigma C protein. (**A**) Time point of duckling experiments. (**B**) Sigma C-specific antibody levels detected by indirect enzyme-linked immunosorbent assay (ELISA) analysis. (**C**) Neutralization antibody levels detected by serum neutralization assay. Each data represents the mean ± SD. ^*^p < 0.05, ^**^P < 0.01 vs. PBS group. ^#^*p* < 0.05 vs. NDRV-TH11 group.

To evaluate whether recombinant sigma C can induce NDRV-specific immune response in ducklings, recombinant sigma C was inoculated into ducklings as described in Materials and methods (shown in Fig. 3A). Blood was collected at weeks 0, 1.0, 2.0, 3.0, and 4.0 after the primary immunization to determine the presence of sigma C-specific and neutralization antibodies. As shown in Fig. 3B, 2.0 weeks after the primary immunization, sigma C-specific antibodies were detected in all the ducklings immunized with recombinant sigma C protein. After the booster immunization, the mean antibody level increased rapidly in sigma C group, and there were significant differences compared with Bac-wt and PBS control groups (*p* < 0.01). Meanwhile, the inactivated NDRV-TH11 vaccine group also exhibited a high antibody level, whereas there was no significant difference between the Bac-sigma C group and NDRV-TH11 group.

Next, serum samples were evaluated in an *in vitro* microneutralization assay to examine their ability to neutralize NDRV TH11 strains in BHK-21 cells. As shown in Fig. 3C, all ducklings immunized with sigma C protein and inactivated NDRV-TH11 vaccine developed neutralizing antibodies at 1.0 weeks after the primary immunization that were significantly higher than that in ducklings in the other two control groups at 3.0 and 4.0 weeks *(p* < 0.01). The average neutralizing antibody titers in the ducklings vaccinated with sigma C was up to 1:45 at 4.0 weeks after the primary immunization, which was higher than inactivated vaccine group (1:32). As expected, none of the ducklings from the Bac-wt and PBS control groups produced detectable neutralizing antibodies during the experiment.

### Cellular immune response in ducklings immunized with sigma C protein

**Figure 4:**
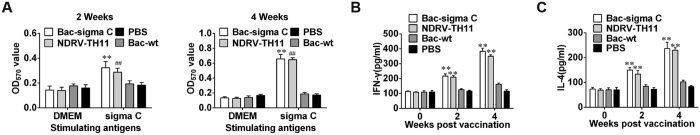
Cellular immune response in ducklings immunized with sigma C. (**A**) The dynamic changes of lymphocyte proliferation in immune response test (A_570_ value). The lymphocyte proliferation response was measured on 2 weeks and 4 weeks after the first immunization. Each data represents the mean ± SD. ^**^P < 0.01 vs. Bac-sigma C group stimulated with DMEM alone. ^##^p < 0.01 vs. NDRV-TH11 group stimulated with DMEM alone. (**B**) The interferon gamma (IFN-γ) concentration in serum harvested from immunized ducklings was measured by enzyme-linked immunosorbent assay (ELISA). (**C**) The interleukin 4 (IL-4) concentration in serum harvested from immunized ducklings was measured by ELISA. Data are the mean ± SD. ^*^P < 0.05, **p < 0.01 vs. PBS group.

To investigate cellular immunity responses induced by sigma C protein, we analyzed the lymphocyte proliferative responses of all ducklings at 2.0 and 4.0 weeks after primary immunization. At 2 and 4 weeks, the proliferative responses were detected in Bac-sigma C group aside from other control groups. On Weeks 2 and 4 after the first vaccination, the values of 2 immunization groups, including Bac-sigma C and NDRV-TH11, were significantly larger than those of Bac-wt and PBS groups (p < 0.01), and in Bac-sigma C group was the highest (Fig. 4A). To further evaluate cellular immunity responses induced by sigma C protein, we analyzed the production of interferon gamma (IFN-γ) and interleukin 4 (IL-4) in duckling serum. As shown in Fig. 4B and C, the levels of IFN-γ and IL-4 in the ducklings vaccinated with sigma C protein were statistically higher than those in other two control groups at 2.0 (*p* < 0.05) and 4.0 weeks (*p* < 0.01) after primary immunization. There are no significant difference between Bac-sigma C group and NDRV-TH11 group. The results indicated that recombinant sigma C protein displayed obvious and intense cellular immune responses in ducklings.

### Protection of ducklings against NDRV challenge

**Figure 5:**
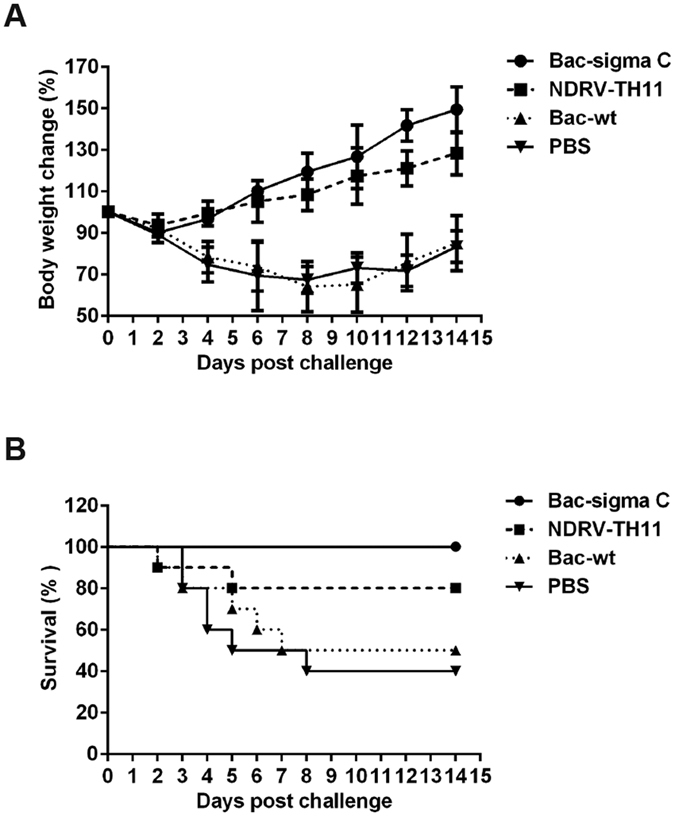
Weight loss and survival rate in ducklings after lethal challenge. (**A**) Weight change was calculated for 14 d after virus challenge and is presented as the mean ± SD percentage of the original body weight. (**B**) Survival curves after challenge. The statistical significance of differences in mortality between groups was determined using the Kaplan-Meier method, and analyzed with a Log-rank (Mantel-Cox) test. For Bac-sigma C vs. PBS, *P* < 0.05; for Bac-sigma C vs. Bac-wt, *p* < 0.05; and for Bac-wt vs. NDRV-TH11, *p* > 0.05.

To investigate whether a higher immune response correlated with better protection, immunized ducklings were challenged at 4.0 weeks after the primary immunization with a dose of NDRV strain TH11 that is lethal in 50% of recipients (LD_50_). All ducklings were housed in an isolation facility and examined for 14 d after the challenge. As shown in Fig. 5A, the two control group ducklings showed serious weight loss and typical symptoms, including inappetence, inability to walk, diarrhea or death, although very slight signs of illness were observed in ducklings immunized with sigma-C. However, 2 of 10 ducklings immunized with inactivated NDRV-TH11 vaccine exhibited typical symptoms and then died. Five and four of the 10 ducklings in PBS group and Bac-wt group reached predefined humane endpoints and were euthanized respectively. No duckling from the Bac-sigma C group was euthanized. As shown in Fig. 5B, control ducklings had an overall survival rate < 50%, and the inactivated NDRV-TH11 vaccine group showed 80% protection. In contrast, the ducklings immunized with sigma C had a survival rate of 100%.

### Detection of NDRV viral load in blood, spleens and livers

**Figure 6:**
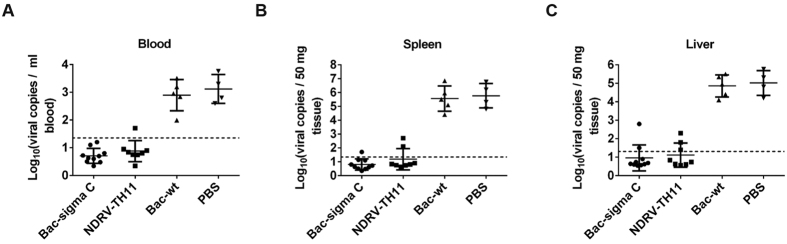
Quantification of viral loads in different tissues of euthanized ducklings using real-time quantitative RT-PCR (qRT-PCR). Samples were collected from ducklings challenged with NDRV-TH11 strain at 14 dpc, and their viral copies were then detected by qRT-PCR. (**A**) Viral loads in blood. (**B**)Viral loads in the spleen. (**C**) Viral loads in the liver. The dotted line marks the positive cut-off. The statistical significance of differences in the virus copies between vaccinated and control groups were determined using One-Way ANOVA and Tukey’s multiple comparison tests between groups. (**p < 0.01 vs. PBS group).

To detect NDRV viral load in the liver, spleen, and blood of ducklings after the virus challenge, real-time quantitative reverse transcription polymerase chain reaction (qRT-PCR) was performed using sigma C gene-specific primers. As shown in Fig. 6, high viral copies were detected in the blood (Fig. 6A), spleens (Fig. 6B) and livers (Fig. 6C) of all ducklings in two control groups. Compared with the control groups, lower viral copies was detected in one of eight blood samples (Fig. 6A), two of eight spleen samples (Fig. 6B) and three of eight livers sample (Fig. 6C) in NDRV-TH11 group ducklings. However, the virus in the blood samples of all ducklings immunized with sigma C vaccine was reduced to undetectable levels on day 14 post challenge (Fig. 6A). Only one of ten in spleen samples (Fig. 6A) and two of ten in liver samples (Fig. 6C) of duckling in Bac-sigma C group were positive for NDRV detection. What’s important, the viral loads in positive samples (liver or spleen) of Bac-sigma C group were also significantly lower compared to two control groups (p < 0.01). These data indicated that vaccination with recombinant sigma C protein could significantly reduce the onset of viremia and decrease virus replication in ducklings.

## Discussion

Since 2000, NDRV disease characterized by severe hemorrhagic-necrotic lesions in the liver and spleen of the sick birds emerged in China and has become an epidemic genotype^7^,^14^,^15^,^16^. NDRV has a wider host spectrum (shelducks, Pekin ducks, wild mallard ducks, Muscovy ducks and goslings) compared with the classical MDRV. The disease causes high morbidity ~5.0 to 50% mortality in ducklings. In a previous studies, we proved that NDRV has low cross-reactivity with classical MDRV and ARV, and that the vaccines against MDRV and ARV cannot protect ducklings from NDRV infection^16^. At present, no vaccine has been available for the prevention of the disease in China.

Inactivated vaccines are frequently used to control many infectious diseases in ducks because they are readily available. In addition, inactivated whole-virus vaccines have been shown to be safe and show high efficacy^30^,^31^. However, there has been little research on the immunity responses and the challenge results caused by inactivated NDRV whole virus vaccine, possibly because NDRV disease is new and prevalent only mainly in China. Chen *et al*. reported that an inactivated NDRV oil-emulsion vaccine can induce strong immune responses and high titers of neutralizing antibodies in shelducks after secondary immunization^32^. Ducklings vaccinated with this oil-emulsion vaccine were effectively protected against the NDRV lethal challenge. However, there are no field data on the results of NDRV inactivated vaccine against natural diseases caused by NDRV. Consequently, despite good experimental results using the NDRV inactivated vaccine in a controlled environment, the effects in the field might not be as successful^33^,^34^,^35^.

Compared to traditional inactivated whole-virus vaccine, recombinant subunit vaccines might provide significant benefits. For example, the production of recombinant subunit vaccines is simple, fast, and economical^36^,^37^. Because subunit vaccines use single components of a pathogen to activate an immune response, the potential advantages of using these vaccines are increased safety and reduced antigenic competition^38^,^39^. For the traditional inactivated vaccines, the complex antigens compete for immune response. In addition, subunit vaccines provide a strategy by which to differentiate infected animals from vaccinated animals^40^. In recent years, many commercial subunit vaccines have been used with good results^41^,^42^,^43^.

ARV genome consists of 10 segments of dsRNA and encodes at least 8 structural (λA, λB, λC, μA, μB, σA, σB, and σC) and 3 to 4 nonstructural (μNS, σNS, P10, and/or P17) proteins. Sigma C protein, a component of the outer capsid layer of the virion, is responsible for host cell attachment^44^ and can induce high levels of type-specific neutralizing antibodies^21^. Moreover, Gaělle *et al*. proved that MDRV sigma C protein could be a candidate for an effective vaccine for protection against field isolates of MDRV^45^. Thus, we speculated that NDRV sigma C protein also has value as a candidate vaccine to prevent and control NDRV. Different expression systems are used to produce recombinant proteins, but the baculovirus expression system is an excellent tool for overexpressing recombinant proteins in insect cells. There are many striking advantages of this system, such as its ability to accommodate a larger exogenous DNA fragment (>30 kb)^46^, relatively easy access to construction and scale production, and accurate post-translational modification. Several commercial vaccines that are based on baculovirus-expressed recombinant proteins have been used^47^,^48^,^49^,^50^,^51^. However, there are no studies on a subunit vaccine based on the baculovirus expression system for the prevention of NDRV infection.

In this study, we successfully constructed recombinant baculovirus expression of the NDRV sigma C gene in Sf9 cells. We also investigated the efficacy of the recombinant sigma C protein as a subunit vaccine against NDRV. Our results demonstrated that immunization with recombinant sigma C protein induces high NDRV-specific lymphocyte proliferation and high levels of IFN-γ (induced by the Th1 cellular response) as well as IL-4 (induced by the Th2 cellular response). In addition, the recombinant sigma C vaccine produced a robust humoral response comprising higher NDRV-specific antibodies and neutralizing antibody titers. More importantly, the recombinant sigma C conferred 100% protection against the lethal challenge in ducklings.

During the development of a vaccine against ARV, research data shown that the level of neutralization antibodies was correlated with protection against ARV infection. In this study, ducklings immunized with recombinant sigma C protein displayed higher NDRV-specific neutralizing antibody titers, most likely attributed to the nature of the sigma C form. In addition, we observed a significantly enhanced cell-mediated immune response in ducklings vaccinated with recombinant sigma C protein. The concentrations of IFN-γ and IL-4 in the ducklings vaccinated with sigma C was to 40 and 65 pg/ml at 4.0 weeks after primary immunization. These data indicated that the recombinant sigma C protein vaccine can induce robust humoral and cellular immune responses in ducklings.

In our previous research, we compared the complete genome sequence between NDRV-TH11 and their counterparts in the other ARV (chicken origin) or MDRV strains^23^. We found NDRV-TH11 has a tricistronic S1 genome segment that encodes p10 (97 amino acids [aa]), a function-unknown protein p18 (162 aa), and sigma C (321 aa), significantly distinct from MDRV, which possesses a bicistronic genome segment that only encodes p10 and sigma C. And the NDRV sigma C sequences shared low levels of identity with ARV (~5.1% nucleotide sequence [nt] identity; ~26.8% aa identity) and MDRV (~36.3% nt identity; ~41.6% aa identity). Maybe this is the reason why the current commercial vaccines against ARVs or MDRVs do not effectively prevent NDRV infection and transmission. Fortunately, the novel viruses (collectively named as NDRV) emerged in China in recent years, including novel duck reovirus^7^,^16^,^52^, the new type MDRV^15^,^53^ and the new type of goose reovirus^6^ showed high sequence identities (Fig. 1B). This makes it possible for using sigma C subunit vaccine originated from NDRV-TH11 strain to control NDRV disease in China. In the follow-up research, we will evaluate the protective effects of our sigma C vaccine against other waterfowl reovirus strains.

## Conclusion

The recombinant NDRV sigma C protein generated using a baculovirus expression system has good antigenicity and immunogenicity in ducklings. The strong immunogenicity makes the recombinant sigma C protein a candidate vaccine against NDRV infection. Further work is required to evaluate the protective effects of the vaccine in field tests.

## Additional Information

**How to cite this article**: Bi, Z. *et al*. Induction of a robust immunity response against novel duck reovirus in ducklings using a subunit vaccine of sigma C protein. *Sci. Rep.* **6**, 39092; doi: 10.1038/srep39092 (2016).

**Publisher's note:** Springer Nature remains neutral with regard to jurisdictional claims in published maps and institutional affiliations.

## Supplementary Material

Supplementary Information

## Figures and Tables

**Table 1:** Primers designed for the construction of pFB-1-sigma C plasmid and detection of viral RNA.

Names	Primer sequences (5′-3′)	Restriction sites
pFastBac-C F	CG**GGATCC**ATGGATCGCAACGAGGTGATACGC	*Bam*HI
pFastBac-C R	GC**TCTAGA**GTGATGATGATGATGATGGCCCGTGGCGAC	*Xba*I
Dia-F	GGAGTCGTCTCACTCCAAGC	
Dia-R	TTTGCGAAGACATGAGCAAC	

Notes: The sequences in bold represent the corresponding restriction site, the underlined sequence indicates the 6 × His tag.
