# Supplementary material for: Induction of a robust immunity response against novel duck reovirus in ducklings using a subunit vaccine of sigma C protein
Source: Sci Rep. 2016 Dec 15;6:39092. doi: 10.1038/srep39092 (PMC5156932; doi:10.1038/srep39092)
Supplement: Supplementary Information [file srep39092-s1.pdf]

Title: Induction of a robust immunity response against novel duck reovirus in ducklings using a subunit vaccine of sigma C protein

Authors: Zhuangli Bi [1, 2, #], Yingqi Zhu [1, 2, #], Zongyan Chen[1], Chuanfeng Li[1], Yong Wang[2], Guijun Wang[2*] and Guangqing Liu[1*]

Affiliations:

[1] Shanghai Veterinary Research Institute, Chinese Academy of Agricultural Sciences, Shanghai 200241, China;

[2] College of Animal Science and Technology, Anhui Agricultural University, Hefei 230036, China.

[*]Corresponding author: Guangqing Liu, Shanghai Veterinary Research Institute, Chinese Academy of Agricultural Sciences, No.518 Ziyue Road, Shanghai 200241, China. Guijun Wang, College of Animal Science and Technology, Anhui Agricultural University, No. 130 West Changjiang Road, Hefei 230036, China. Tel.:+86-21-34293498; Fax: + 86-21-34293498; E-mail address: liugq@shvri.ac.cn; Wangguijun@ahau.edu.cn

[#]Zhuangli Bi and Yingqi Zhu contributed equally to this paper.

Supplementary Materials:

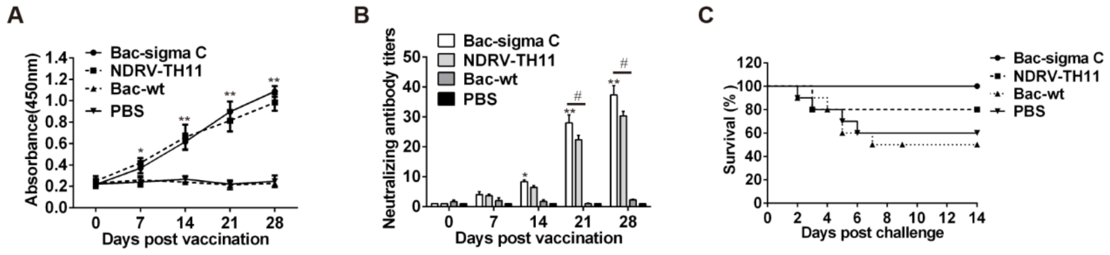

Supplementary Figure 1. Antibody response and survival rate in ducklings immunized with recombinant sigma C protein (the second replicate experiment). (A) Sigma C-specific antibody levels detected by indirect enzyme-linked immunosorbent assay (ELISA) analysis. (B) Neutralization antibody levels detected by serum neutralization assay. Each data represents the mean ± SD. *, p < 0.05, **, P < 0.01 vs PBS group. $^{\#}$, p < 0.05 vs NDRV-TH11 group. (C) Survival curves after challenge. The statistical significance of differences in mortality between groups was determined using the Kaplan-Meier method, and analyzed with a Log-rank (Mantel-Cox) test. For Bac-sigma C vs PBS, P < 0.05; for Bac-sigma C vs Bac-wt, p < 0.05; and for Bac-wt vs NDRV-TH11, p > 0.05.

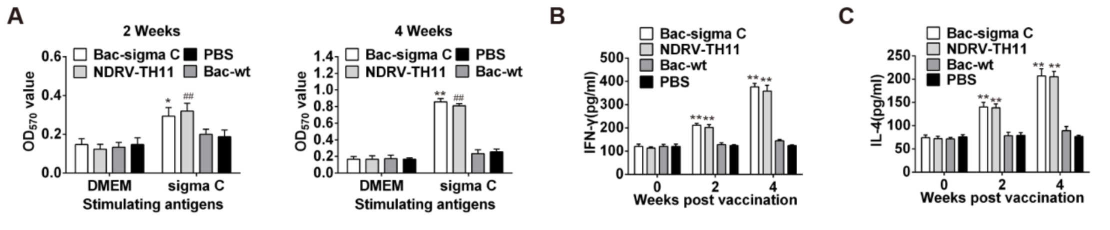

Supplementary Figure 2. Cellular immune response in ducklings immunized with sigma C (the second replicate experiment). (A) The dynamic changes of lymphocyte proliferation in immune response test (A_{570} value). The lymphocyte proliferation response was measured on 2 weeks and 4 weeks after the first

immunization. Each data represents the mean ± SD. *, P < 0.05, **, P < 0.01 vs Bac-sigma C group stimulated with DMEM alone. ##, p < 0.01 vs NDRV-TH11 group stimulated with DMEM alone. (B) The interferon gamma (IFN-γ) concentration in serum harvested from immunized ducklings was measured by enzyme-linked immunosorbent assay (ELISA). (C) The interleukin 4 (IL-4) concentration in serum harvested from immunized ducklings was measured by ELISA. Data are the mean ± SD. **, $p < 0.01$ vs PBS group.

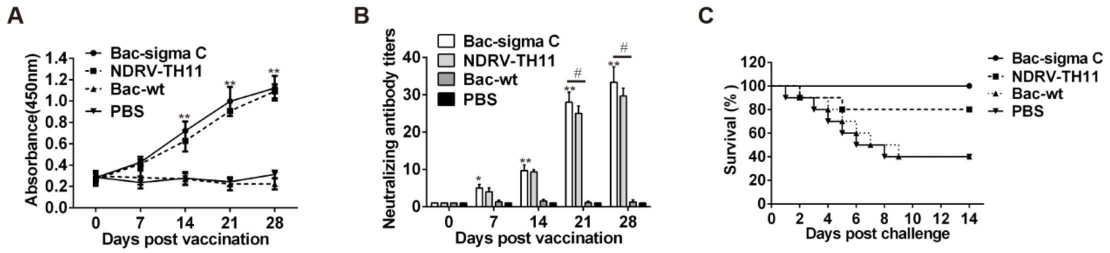

Supplementary Figure 3. Antibody response and survival rate in ducklings immunized with recombinant sigma C protein (the third replicate experiment). (A) Sigma C-specific antibody levels detected by indirect enzyme-linked immunosorbent assay (ELISA) analysis. (B) Neutralization antibody levels detected by serum neutralization assay. Each data represents the mean ± SD. *, $p < 0.05$, **, $P < 0.01$ vs PBS group. #, $p < 0.05$ vs NDRV-TH11 group. (C) Survival curves after challenge. The statistical significance of differences in mortality between groups was determined using the Kaplan-Meier method, and analyzed with a Log-rank (Mantel-Cox) test. For Bac-sigma C vs PBS, $P < 0.05$; for Bac-sigma C vs Bac-wt, $p < 0.05$; and for Bac-wt vs NDRV-TH11, $p > 0.05$.

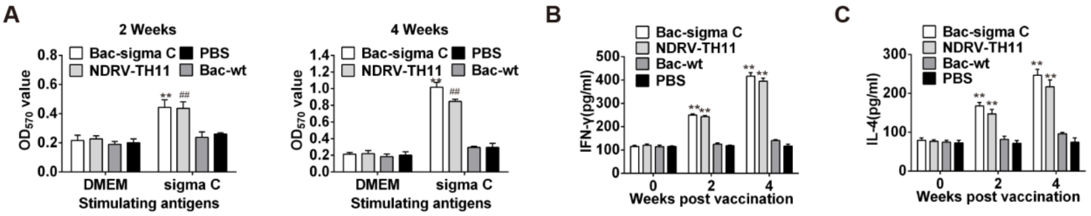

Supplementary Figure 4. Cellular immune response in ducklings immunized with sigma C (the third replicate experiment). (A) The dynamic changes of lymphocyte proliferation in immune response test (A_{570} value). The lymphocyte proliferation response was measured on 2 weeks and 4 weeks after the first immunization. Each data represents the mean ± SD. **, P < 0.01 vs Bac-sigma C group stimulated with DMEM alone. $^{##}$, p < 0.01 vs NDRV-TH11 group stimulated with DMEM alone. (B) The interferon gamma (IFN-γ) concentration in serum harvested from immunized ducklings was measured by enzyme-linked immunosorbent assay (ELISA). (C) The interleukin 4 (IL-4) concentration in serum harvested from immunized ducklings was measured by ELISA. Data are the mean ± SD. **, *p* < 0.01 vs PBS group.
